# Dietary Intake of Polyphenols Enhances Executive/Attentional Functioning and Memory with an Improvement of the Milk Lipid Profile of Postpartum Women from Argentina

**DOI:** 10.3390/jintelligence10020033

**Published:** 2022-05-31

**Authors:** Agustín Ramiro Miranda, Mariela Valentina Cortez, Ana Veronica Scotta, Elio Andrés Soria

**Affiliations:** 1 Health Sciences Research Institute, National Scientific and Technical Research Council, Córdoba 5014, Argentina; armiranda@fcm.unc.edu.ar (A.R.M.); mvcortez@fcm.unc.edu.ar (M.V.C.); avscotta@fcm.unc.edu.ar (A.V.S.); 2 School of Phonoaudiology, Faculty of Medical Sciences, National University of Córdoba, Córdoba 5014, Argentina; 3 Chair of Cell Biology, Histology and Embryology, Institute of Cell Biology, National University of Córdoba, Córdoba 5014, Argentina

**Keywords:** breast feeding, diet, language tests, lipids, mental health, oxidative stress, polyphenols, Rey Auditory Verbal Learning Test, Stroop Test, Wisconsin Card Sorting Test

## Abstract

Puerperium may lead to memory and executive/attentional complaints that interfere with women’s daily life. This might be prevented by dietary compounds, such as neuroprotective polyphenols. Their bioactivity depends on their effects on lipid metabolism in different tissues, such as the brain, fat, and breast. Thus, a polyphenol-related cognitive improvement may be associated with changes of lipids in human milk, which are key for infant neurodevelopment. A cross-sectional study was conducted on 75 postpartum women from Córdoba (Argentina), involving several neuropsychological tests. Diet was registered to identify polyphenol intake and food pattern adherence, with sociodemographic and other psychological variables (insomnia, stress, subjective cognitive complaints) being also studied. Triacylglycerols, cholesterol, and their oxidative forms were analyzed as milk biomarkers. Multivariate statistical methods were applied. Results confirmed that women who consumed polyphenols presented better executive/attentional performance (i.e., higher correct responses, conceptual level responses, complete categories, verbal fluency; lower attentional interferences, and perseverative errors) and word retention with lower interference. Polyphenols were positively associated with milk lipids, which were higher in women with better cognition. Furthermore, they had lower oxidized triacylglycerols. In conclusion, polyphenolic intake during postpartum may improve executive/attentional functioning, memory, and milk lipid profile.

## 1. Introduction

Cognitive functioning during puerperium depends on neuropsychological adaptations with important psychosocial consequences for women, which require special attention in postpartum health care (Carrizo et al. 2020). Furthermore, women’s vulnerability was triggered by the impact on daily life derived from the COVID-19 pandemic, leading to stress, postpartum depression, insomnia, and cognitive impairment (Miranda et al. 2021b; Miranda et al. 2022a).

Executive/attentional functioning involves several processes, such as inhibition, planning, mental shifting, decision taking, updating, and dual-task coordination (Gilsoul et al. 2019; Tull et al. 2012). Puerperium also leads to memory adaptations, involving changes in neural networks and functional connectivity (Bak et al. 2020; Shin et al. 2018). Specific instruments are necessary to assess these changes. Complaints on these spheres compromise different aspects of women’s lives, such as nurture, breastfeeding, work, and study (Miranda et al. 2020b; Scotta et al. 2022). These concerns involve a persistent decline in memory, linguistic, attentional, and executive domains in women with previously normal frontal-executive functioning (Hohman et al. 2011; Mendonça et al. 2016). Thus, it is necessary to study preventative factors that support effective mental health measures.

Among these factors, nutrition is a crucial multitarget determinant of human health and well-being. In this sense, the diet provides several bioactive compounds that can prevent neuropsychological compromise. Polyphenols are promising candidates because they exert a wide range of neurotrophic effects (Miranda et al. 2018). These are heterogeneous compounds grouped into different chemical families, such as flavonoids (e.g., flavonols), and non-flavonoids (e.g., phenolic acids). They are present in plant foods, such as glycosides or aglycones (Singla et al. 2019). Although their bioactivity was thoroughly documented, the relevance of polyphenols as part of a habitual diet is still unclear due to the complexity of human nutrition and metabolism (Luca et al. 2020). Nonetheless, previous research suggested that the dietary intake of certain polyphenol families enhances memory during postpartum (Miranda et al. 2021a). Thus, HJ-Biplot, an innovative methodological approach in nutritional epidemiology, will be applied herein. This statistical method was recently developed to evaluate polyphenols comprehensively and their impact on lactating women (Miranda et al. 2022b).

Dietary polyphenols regulate lipid metabolism, which underlies several molecular mechanisms in the brain, fat, liver, endocrine system, muscles, and gut (Matacchione et al. 2020). Polyphenols are responsible for the Mediterranean diet’s benefits, such as preventing metabolic and neurological diseases that involve low-grade inflammation, oxidative stress, and anomalies in lipid metabolism (Milošević et al. 2021). Nonetheless, their involvement in the mammary gland, which may present unique responsiveness to these compounds, is partially unknown. Certain antioxidant effects on human milk lipids were reported (Poniedziałek et al. 2018; Valls-Bellés et al. 2021). Although the physiological mechanisms remain unclear, they may protect against peroxidative damage-related lipid degradation, upregulate gene expression related to lipid metabolism in the mammary gland, modulate neuroendocrine pathways, and enhance milk lipid clearance (Codini et al. 2020; Downing et al. 2017; Lin et al. 2018). Thus, additional biochemical studies are still needed because lipids are indispensable nutrients for healthy child development (Brink and Lönnerdal 2020).

Polyphenols target molecular pathways of lipid metabolism that are common to the brain and breast (Ehrmann et al. 2002; Senthil et al. 2021). Therefore, changes in the milk content of triacylglycerols and cholesterol may reflect a more complex internal response, which can participate in a cognitive improvement. Moreover, maternal behavior and milk production share neuroendocrine mediators, which determine lactating physiology and cognition (Jurek and Neumann 2018; Kiełbasa et al. 2021). Thus, this work aimed to establish the effects of dietary polyphenols on lactating women, based on the hypothesis that polyphenol intake during postpartum enhances executive/attentional functioning and memory in association with a quantitative and qualitative improvement of the human milk lipids.

## 2. Materials and Methods

### 2.1. Study Design and Participants

This cross-sectional, analytical study was performed on 75 lactating women from Córdoba province (Argentina), who were interviewed face-to-face by health professionals trained in neuropsychology and nutrition assessment in primary care units of the public health system, the Maternal-Neonatal Hospital “Prof. Dr. Ramón Carrillo” and the Medical Sciences’ facilities of the National University of Cordoba. The inclusion criteria were: adult (≥18 years old), Córdoba inhabitant, postpartum (first six months), and breastfeeding. Exclusion criteria were: pregnancy, alcohol and drug abuse, currently active disease, neuropathology, and psychological conditions (e.g., depression).

Women signed an informed consent to be included voluntarily in this epidemiological study, approved by the Research Ethics Committee of the National Hospital of Clinics (National University of Cordoba), in accordance with the Declaration of Helsinki and current national legislation. Approval codes were RENIS-IS000548, RENIS-IS001262, RENIS-IS002045 (national registration), REPIS-145, REPIS-2654, and REPIS-5554 (province registration).

### 2.2. Interview

The assessments were carried out on working days (between 8 a.m. and 6 p.m.) and took place in a quiet test room at a hospital, faculty, or private setting. Participants were individually tested by trained technicians in neuropsychology (for the cognitive assessment) and in nutrition (for the nutritional assessment) in a single session lasting, on average, 120 min. Regarding the cognitive assessment, the participants responded to the tasks in the same order: first the participants answered the Stroop task, then the Rey Auditory Verbal Learning Test, then they were assessed with the Wisconsin Card Sorting Test, and finally the Verbal Fluency Tasks. The rooms were sound-proofed and light controlled, had adequate ventilation and temperature, and the women were provided with comfortable furniture (chair and desk), as well as a babysitter to take care of their children during the assessment. The neuropsychological assessment was performed before the nutritional assessment.

#### 2.2.1. General Evaluation

The following sociodemographic data were collected using an ad hoc questionnaire: age (years), educational level (<12 or ≥12 years of formal instruction), employment (formally employed or informally employed/unemployed), marital status (single or in a couple), number of births (primiparous or multiparous), gestational age at delivery (<37 weeks -preterm or ≥37 weeks -term-), time of milk sampling (08.00 to 12.00, 12.00 to 16.00, or 16.00 to 20.00), practice of exclusive breastfeeding (yes or no), and postpartum duration (days). In addition, the type of physical activity, minutes and times per week, were registered to calculate the metabolic equivalents (MET) (Roman-Viñas et al. 2010).

#### 2.2.2. Cognitive Assessment

All of the participants were neuropsychologically assessed with a set of validated tests to evaluate attention, language, memory, and executive functions.

##### Attention

The Stroop Color and Word Test (SCWT- Stroop 1935) is an instrument widely used in clinics and research that provides measures of inhibition, selective attention, processing speed, and mental flexibility (Rivera et al. 2015). Each one of its three sheets has 100 randomly organized elements in five columns. The first two sheets represent the “congruent condition” in which the participants should read the names of the “red”, “green”, and “blue” colors printed in black ink (first sheet “word”-W-) and name the ink color (red, green, and blue) in which “xxxx” (second sheet, “color”-C-) are printed. On the contrary, the “incongruous condition” is presented in the third sheet (“word-color”-WC-), in which the words of color are printed with a different color ink on the name of the color (for example, the word “blue” is printed with red ink) (Scarpina and Tagini 2017). For each sheet, participants were instructed to read from top to bottom and from left to right as quickly as possible, and the score derived from the number of items correctly appointed in 45 s. An interference rate (I) was calculated as I = WC − [(W × C)/(W + C)] (Rivera et al. 2015; Scarpina and Tagini 2017). Successful execution on the third page requires that subjects intentionally concentrate their attention to an aspect of the stimulus (print color), while inhibiting attention from the other (meaning of the word). SCWT has good psychometric properties for Latin American Spanish speakers (Rodríguez Barreto et al. 2016).

##### Memory

The Rey Auditory Verbal Learning Test (RAVLT) for Spanish speakers was used to assess episodic memory (Ferreira Correia and Campagna Osorio 2014). This widely known test, developed by Rey (1958), is considered easy and quick to administer. RAVLT consists of presenting orally a list of 15 unrelated words in five consecutive trials (Burin et al. 2007). Then, a new list is read with 15 distractors (List B), and the person must remember them immediately. Next, participants are required to remember the words of the first list (Trial A6-postinterference free recall-). After 30 min, the participant must again remember the words of the first list (Trial A7-long term delayed free recall-), and finally identify them on a printed sheet with 75 words (visual recognition). For this study, a set of traditional and novel scores were obtained from RAVLT (Alviarez-Schulze et al. 2022; Miranda et al. 2021a; Tyagi et al. 2021).

##### Executive Functions

The manual version of the Wisconsin Card Classification Test (WCST) was used to assess executive functioning (De la Cruz 2001; Heaton et al. 1993). This task-switching instrument originally developed by Berg (1948), consists of 4 reference cards and 128 response cards, which are divided into two decks of 64 cards each. The cards contain geometric figures, which vary in shape (crosses, circles, triangles, or stars), color (red, blue, yellow, or green), and number (one, two, three, or four). The participant must match the cards with one of the four reference cards. The sorting criterion is not indicated, and after the subject’s response, the evaluator indicates whether the card was correctly or incorrectly matched, but still does not reveal the principle of correct classification. Therefore, this forces the subject to repeat the responses until he/she discovers the classification rule. Once the subject has correctly classified ten consecutive cards, the criterion changes without prior notice, so that the subject must modify their classification criteria to identify the new one. Thus, this instrument is widely used to measure higher-level cognitive processes related to the prefrontal cortex (e.g., attention, perseverance, working memory, abstract reasoning, cognitive flexibility, inhibitory control, and set shifting) (Faustino et al. 2021). For this purpose, traditional WCST scores were used for the analysis of executive functions (Faustino et al. 2020). This instrument proved to be reliable and valid for the study of executive functions with local normative data for Spanish speakers (Miranda et al. 2020a).

##### Language

Linguistic functioning was evaluated using four verbal fluency tasks (VFT): two phonological tasks with direct instructions (initial letter P and F), one semantics (animals), and one phonological with indirect instructions (words without “A”- excluded letter A-). VFT was systematized by Borkowski et al. (1967). The application of the VFT followed the procedure proposed by Marino and Alderete (2010). The time allotted for each test was one minute. All of the words were recorded on a spreadsheet and each correct answer was scored with one point. Intrusions (words that do not belong to the required semantic field or do not comply with the phonological instruction) and perseverations (the repetition of a previous response) were considered incorrect (Lezak et al. 2012). VFTs are a useful tool to evaluate different capacities and cognitive functions related to language, such as lexical-semantic memory and executive/attentional processes (Cardoso Silva de Souza et al. 2020). VFTs have adequate psychometric properties and there are normative data available for several VFTs for Spanish speakers (Marino and Alderete 2010).

#### 2.2.3. Nutritional Assessment

A validated food frequency questionnaire (FFQ) was applied to record the usual diet during the last 12 months. The FFQ comprises 127 foods available in the country, grouped according to their nutritional profile and origin. Questions were about frequency (times per month/week/day, as appropriate) and usual portion size of each food consumed (three categories: small, medium, and large). This instrument has shown adequate levels of validity and reproducibility for the studied population (Cortez et al. 2021). A validated photographic atlas was used, based on the standard portion sizes in Argentina to help participants describe the amount of food consumed. Next, data were analyzed using the Phenol-Explorer database (version 3.6) (Rothwell et al. 2013). This database provides values for more than 500 different polyphenols in more than 400 foods in different forms, including raw, cooked, and processed foods. The intake was calculated as mg/day. Only polyphenols with a medium daily intake ≥5 mg/d were considered for the analyses (Kesse-Guyot et al. 2012). This cut off point was previously demonstrated to present health effects (Miranda et al. 2022b).

Nutritional assessment included anthropometric measures: self-reported height (meters) and weight (kilograms) were recorded to calculate body mass index (BMI); and body fat percentage (BFP) was measured by hand-held bioelectrical impedance analysis (Cortez et al. 2021).

### 2.3. Milk Sampling and Lipid Measurement

The sampling protocol of Cortez and Soria (2016) was followed. Ten mL of human milk was delivered by each woman by manual expression into sterile collection bottles. Samples were immediately stored at −20 °C for transportation, and then at −80 °C to avoid lipid deterioration by enzymatic activity until the analyses (George et al. 2018). Triacylglycerols and cholesterol were measured by spectrophotometry at 540 nm, using a Promega’s GloMax Multi Microplate Multimode Reader (USA).

#### 2.3.1. Triacylglycerols

Triacylglycerols were estimated by a widely used enzymatic/chromogenic method provided by a GT Lab commercial kit under the manufacturer’s instructions (GPO/PAP, Triglicéridos Liquid PlusTM, GT Lab, Rosario, Argentina), who established the corresponding analytical conditions. The assay involves the following enzymes: lipase (which hydrolyzes triglycerides to glycerol and fatty acids); glycerol kinase (which phosphorylates to glycerol, forming glycerol-1-phosphate); glycerol phosphate oxidase (which oxidizes to glycerol 1-phosphate, forming dihydroxyacetone-phosphate and hydrogen peroxide); and peroxidase (which in the presence of 4-aminophenazone forms a pink color quinonimine from hydrogen peroxide) (Kumari et al. 2020). Briefly, 3 parts of milk were mixed with 300 parts of a reactant solution for 5 min at 37 °C. Triacylglycerol content per liter (g/L) was then calculated by measuring the reaction at 540 nm and using the corresponding standard.

#### 2.3.2. Cholesterol

Milk cholesterol concentration was estimated using an enzymatic/colorimetric method provided by a GT Lab kit, following the manufacturer’s instructions (CHOD/PAP, Colesterol Liquid Plus^TM^, GT Lab, Rosario, Argentina), who established the corresponding analytical conditions. Firstly, cholesterol esters are hydrolyzed by cholesterol esterase. Subsequently, the free cholesterol is oxidized by cholesterol oxidase to Δ4-cholestenone. Under peroxidase action, the hydrogen peroxide produced in the previous reaction oxidizes the chromophore 4-aminophenazone, in the presence of phenol, to the red dye compound 4-(p-benzoquinone-monoimino)-phenazone (Kumari et al. 2020). It was measured at 540 nm to calculate mg/L using the corresponding standard, after 3 parts of milk and 300 parts of a reactant solution were mixed for 5 min at 37 °C.

#### 2.3.3. Oxidized Lipids

Lipoperoxides are products of the oxidative damage of fatty acids and cholesterol in cell membranes and lipoproteins (Elisia and Kitts 2011). To determine their concentration in human milk, a commercial kit was used, according to the manufacturer’s instructions (PeroxiDetect^TM^, Sigma-Aldrich, Saint Louis, MO, USA), who established the corresponding analytical conditions. One part of HM, 0.1 parts of 25 mM ferrous ammonium sulfate (prepared in 2.5 M sulfuric acid), and 10 parts of a reactant (125 μM xylenol orange with 4 mM butylated hydroxytoluene) were mixed for 30 min at 37 °C to reveal the optical density of lipoperoxides at 540 nm per liter of milk (OD/L). For the lipid hydroperoxides, the measuring procedure was similar to that of lipoperoxides, but the reactant was replaced by 125 μM xylenol orange in 100 mM sorbitol. Given that triacylglycerols are the form of storage and transportation of the principal targets of lipid peroxidation (polyunsaturated fatty acids), the quotient between lipoperoxides and triacylglycerols were calculated as OD/g (Pérez-Rodríguez et al. 2015). On the other hand, oxysterols are oxidized cholesterol derivatives that can be generated in the human organism through different oxidation processes. The quotient between the lipoperoxides and cholesterol indicated non-polar oxysterols (e.g., esterified cholesterol), while the hydroperoxides/cholesterol quotient indicated polar oxysterols (e.g., non-esterified cholesterol or lipoprotein) (Brown and Jessup 2009), with the level of oxysterols being reported as OD/mg.

### 2.4. Other Measures

#### 2.4.1. Maternal Insomnia

The Spanish version of the Insomnia Severity Index (ISI) was used to evaluate the nature, severity, and impact of insomnia during the last month (Morin et al. 2011). This seven item self-report instrument is a traditional questionnaire used in insomnia research (Manzar et al. 2021). Each item was responded using a five-point Likert scale (0 to 4, where 0 = no problem and 4 = very severe problem) and the scores were transformed on a scale ranging from 0 to 28, with the highest scores indicating more severe insomnia and impact on quality of life. In the current study, the ISI showed good reliability (McDonald’s omega coefficient = 0.733).

#### 2.4.2. Maternal Adherence to Dietary Patterns

Adherence to dietary patterns was evaluated using dietary indices for Argentinian lactating women. Adherence was calculated using three indices, based on the patterns identified by Cortez et al. (2021), which includes the following:

- Macro-nutritional Dietary Index (MDI): Snacks; processed meats; legumes; dairy products; cheese; whole grains; and candies;
- Phytochemical Dietary Index (PDI): vegetables A (5% of carbohydrate content); vegetables B (10% of carbohydrate content); fruits; and fatty fruits;
- Energetic Dietary Index (EDI): vegetables C (20% of carbohydrate content); animal fats; vegetable oils; sugary drinks; and refined grains.

The dietary intake of each group was divided into deciles, with each decile being scored from 1 (lowest) to 10 (highest), whereas no intake received 0 points. The scoring system was adjusted for food groups with low intake frequency (i.e., snacks). Finally, points were totaled: MDI ranged from 0–41 points; PDI from 0–35; and EDI from 0–39, with higher scores indicating greater dietary pattern adherence.

In order to evaluate the quality of fats, carbohydrates, and proteins in the maternal diet, the Fat Quality Index (FQI) and the Protein to Carbohydrate Ratio (PCR) were calculated (Miranda et al. 2022b).

#### 2.4.3. Subjective Memory Complaints

Participants responded to a Spanish version of the Memory Failures of Everyday questionnaire (MFE) (Lozoya-Delgado et al. 2012), which explores subjective memory complaints and related cognitive processes, such as attention, perceptual recognition, and language (Rodríguez-Blázquez et al. 2022). This instrument consists of 30 items about memory failures during situations and activities that take place in everyday life, which must be rated using a five-point Likert scale (0 to 4, 0 = never and 4 = always). In this study, MFE showed excellent reliability (McDonald’s omega coefficient = 0.924).

#### 2.4.4. Subjective Executive Complaints

The Executive Complaint Questionnaire (ECQ) was used (Mías 2010). This questionnaire explores the subjective complaints of executive functions. The questionnaire has 15 items and participants must judge to what extent a series of daily life behaviors concerning mental functioning occur (Miranda et al. 2020c). Each item was rated on a five-point Likert scale (0 to 4, where 0 = never and 4 = always). In this work, it was found that the ECQ has a very good reliability (McDonald’s omega coefficient = 0.821).

### 2.5. Statistical Analyses

Statistical analyses were performed using Infostat (version 2020, InfoStat Group, Córdoba, Argentina) and Stata (version 15, StataCorp, College Station, TX, USA). Mean, standard deviation (SD), median, 25th, and 75th percentiles were calculated for numerical variables, and percentages for categorical ones. Skewness (S) and kurtosis (K) were used to assess the distribution of the neuropsychological scores. McDonald’s omega coefficient was estimated to assess the reliability of self-report questionnaires.

HJ-Biplot multivariate analyses were performed to discover the correlations between polyphenols, as well as cognitive scores. This technique provides a graphical representation of the rows and columns of a data matrix in a low-dimensional subspace where their relative positions are interpretable (Frutos Bernal et al. 2020). The markers are obtained from the singular value decomposition of the data matrix, where the rows represent individuals (women), and the columns variables (polyphenols or cognitive scores), so that both sets of markers can be overlaid in the same reference frame with the highest quality of representation. The HJ-Biplot graphic representation must be interpreted based on the following criteria (Carrasco et al. 2019):

- The distances between points are interpreted as an inverse function of their similarities;
- The vector length approximates the variable standard deviation;
- The cosines of the angles between the vectors approximate the correlations between them, and the cosines of the angles between vectors and axes approximate the correlations between both;
- The order of the orthogonal projections of the points onto a vector approximates the order of those points in that vector.

Clustering analyses, based on coordinates using the K-means method, were conducted in the HJ-Biplot models (Nieto-Librero et al. 2017). This multivariate classification technique enables the detection and description of clusters of subjects or homogeneous variables, based on the values observed within a heterogeneous set, trying to achieve the maximum possible homogeneity in each group and the significant differences between them. Similarity calculation between the centroids and data points (i.e., women) was based on cosine similarity (Carrasco et al. 2019). Contributions and qualities of representation (QLR) for each variable and cluster on the factorial plane were calculated as data fit measures. It was shown that the HJ-Biplot is a valid tool for the study of nutrition and cognitive functioning (Miranda et al. 2022b; Cadavid Ruiz et al. 2018). The HJ-Biplot analysis was performed using the MULTBIPLOT software package (multivariate analysis using BIPLOT) (Vicente-Villardón 2015).

**Figure 1:**
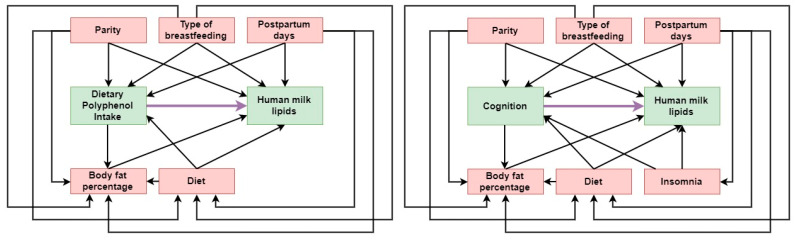
Directed acyclic graph of the relationship among polyphenol intake, cognition, and human milk lipids in postpartum Argentinian women. Green squares indicate the independent (dietary polyphenol intake and cognition) and dependent variables (human milk lipids); red squares indicate minimal sufficient adjustment variables; the bold arrow indicates the relationship between the independent and dependent variables; thin arrows indicate other causal relationships.

Analyses of covariance (ANCOVA) with Fisher’s post hoc comparisons were used to test the differences in cognitive performance among the polyphenolic clusters. In addition, ANCOVA was used to analyze the effect of dietary polyphenols and cognitive status on lipids in human milk. All of the models were adjusted by potential confounders (β). The sets of potential confounders for each model were selected according to directed acyclic graphs, using the criterion of minimal sufficient adjustment for direct effects (Figure 1) (Miranda et al. 2021a). Values of 0 and 1 were adopted to establish the absence and presence, respectively, of the covariates introduced in the ANCOVA models, which combines an analysis of variance to the data and a multiple linear regression to the data to eliminate the effect of the covariates. Moreover, a dichotomous factor can be entered into a regression equation by formulating a dummy regressor with a 0/1 codification (Fox 2015). The use of several covariates reduces the variability of the data, thus increasing the statistical power, with models being fitted appropriately in accordance with sample size and prior criteria (Shieh 2020). Coefficients of determinations of the model (R^2^), means, standard errors, *p*-values (for comparisons), and Cohen’s d coefficients were reported. Effect sizes were calculated using Cohen’s d, which were interpreted as small (≥0.20), medium (≥0.50), large (≥0.80), and very large (≥1.30). Finally, post hoc analyses showed that the sample size in this study achieved more than 80% power with an alpha of 0.05, taking 8 predictors in the ANCOVA models and size effects of 0.45. The homogeneity of variance among the groups was tested by Levene’s test (Mishra et al. 2019).

## 3. Results

### 3.1. Sample Characteristics

Maternal age averaged 29.92 years (SD = 6.13). Most women were in a couple (93.33%), and had finished at least 12 years of formal education (75.66%). Unemployment or informal employment affected 54.66% of the participants. Regarding reproductive variables, the mean postpartum days were 95.04 (SD = 52.46), with a pregnancy duration of 38.37 weeks (SD = 2.05). Cesarean sections were presented in 55% of the sample, and 60% of the women were multiparous. The percentage of participants who practiced exclusive breastfeeding was 53.33%. In relation to the nutrition status, women had a BMI of 24.79 (5.62) Kg/m^2^ and a BFP of 28.88 (7.00) %, while they exhibited 384.96 (SD = 686.33) MET of physical activity. Means of dietary adherence were similar: 25.25 (SD = 6.85) for MDI, 21.89 (SD = 7.18) for PDI, and 22.81 (SD = 7.71) for EDI (Table 1).

Table 2 describes the WCST performances. All of the scores showed levels of skewness and kurtosis ± 3, suggesting a normal distribution, except for trials to complete the first category, and the percentage of perseverative errors. Similarly, most scores in the RAVLT showed a normal approximation, except for the amount of intrusion errors, percentage of forgetting, and retroactive interference (Table 3). All of the SCWT and VFT scores showed acceptable values for skewness and kurtosis (Table 4).

### 3.2. Diet Characteristics

**Figure 2:**
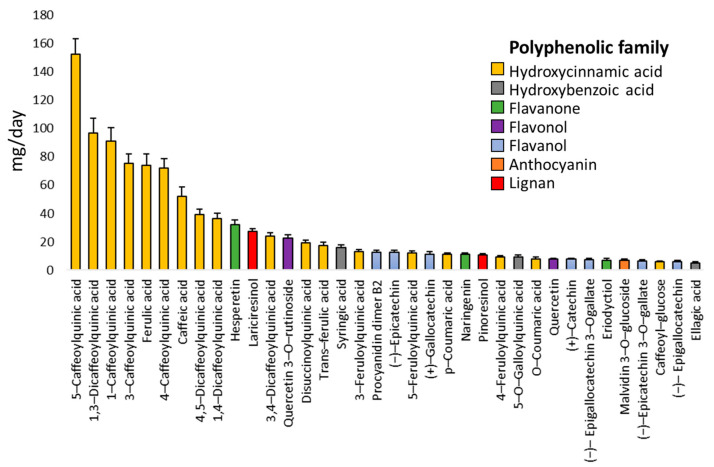
Daily dietary polyphenolic intake in postpartum women from Córdoba, Argentina (*n* = 75), with data being expressed as mean and standard errors.

**Figure 3:**
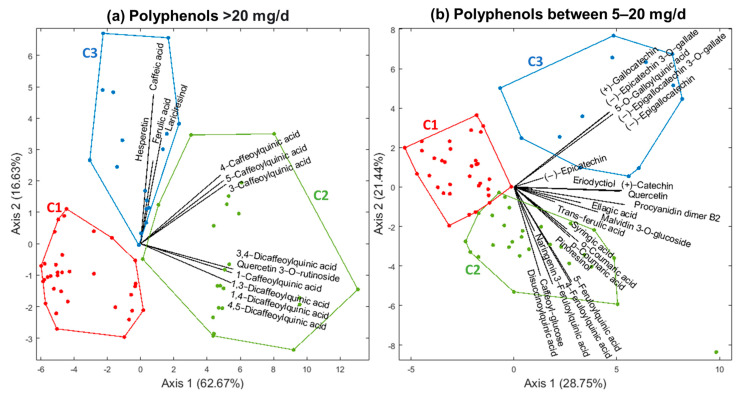
HJ-Biplot representation (axis 1–2) for clusters of polyphenols consumed above 20 mg/d (**a**) and between 5 and 20 mg/d (**b**) by Argentinian postpartum women.

Thirty-six polyphenolic compounds were consumed at levels above 5 mg/d (Figure 2), including the following families: hydroxycinnamic acids (50%); flavanols (19.44%); flavanones (8.33%); hydroxybenzoic acids (8.33%); flavonols (5.55%); lignans (5.55%); and anthocyanin (2.77%). Figure 3 shows the HJ-Biplot graphics to visually represent the polyphenolic intake models in the factorial plane 1–2. The first model included those polyphenols consumed above 20 mg/d, and explained the 79.3% of variance (Figure 3a). These compounds showed adequate levels of contributions to the plane 1–2, and were represented with adequate quality (QLR > 200) (Supplementary Materials: Table S1). Three women clusters were identified in this model: C1 (women with lower polyphenolic intake, *n* = 34); C2 (women with higher intake of different hydroxycinnamic acids and one flavonol, *n* = 24); and C3 (women with higher intake of two hydroxycinnamic acids, one lignan, and one flavanone, *n* = 17). The qualities of representation for each cluster on the plane 1–2 exceed the value of 95%. 

On the other hand, Figure 3b shows the HJ-Biplot representation on the main first plane for the polyphenols consumed between 5 and 20 mg/d, which explained the 50.19% of variance. Most of these compounds showed adequate representation qualities, except for naringenin, eriodyctiol, syringic, ellagic acid, and trans-ferulic acid (QLR < 150) (Supplementary Materials: Table S2). Three clusters were formed with qualities of representation above 90%: C1 (women with lower polyphenolic intake, *n* = 33); C2 (women with higher intake of different hydroxycinnamic acids, one hydroxybenzoic acid, and one lignan, *n* = 29); and C3 (women with higher intake of different flavanols and one hydroxybenzoic acid, *n* = 13).

### 3.3. Effects of Polyphenols on Women’s Cognition

**Figure 4:**
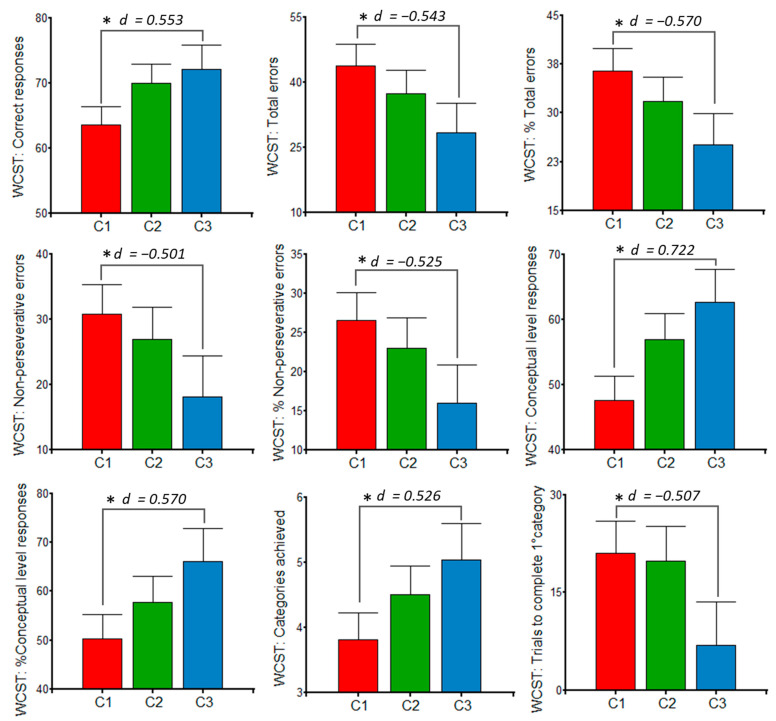
Wisconsin Card Sorting Test (WCST) scores according to polyphenolic intake clusters (>20 mg/d). ANCOVA models were carried out with Fisher’s post hoc contrast and adjusted by educational level, postpartum days, parity, dietary indices (macronutritional, phytochemical, and energetic), and insomnia. C1: *n* = 34; C2: *n* = 24; C3: *n* = 17. *D* = Cohen’s d for effect size; * *p* < 0.05.

For polyphenols > 20 mg/d, women from C3 had a significantly higher number of correct responses, conceptual level responses, percentage of conceptual level responses, and categories achieved in the WCST, with respect to C1. Moreover, C3 had lower total errors, percentage of total errors, non-perseverative responses, percentage of non-perseverative responses, and trials to complete the first category. All of these significant differences on the WCST scores presented moderate effect sizes (Cohen’s d ≥ 0.50) (Figure 4).

**Figure 5:**
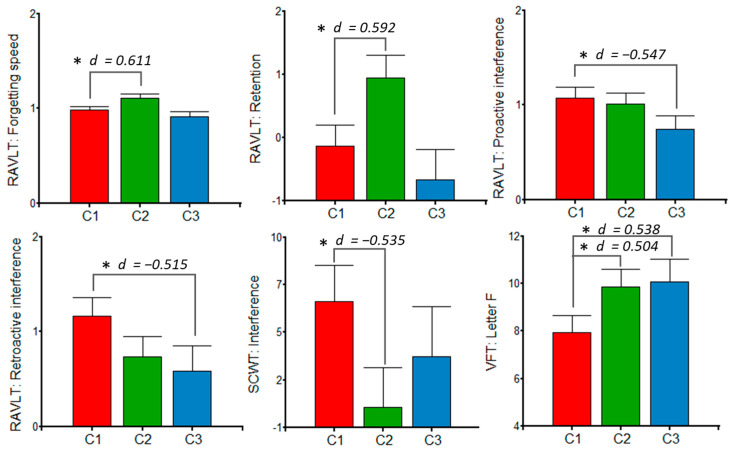
Postpartum women’s performance in the Rey Auditory Verbal Learning Test (RAVLT), Stroop Color and Word Test (SCWT), and Verbal Fluency Task (VFT) according to polyphenolic intake clusters (>20 mg/d). ANCOVA models were adjusted by educational level, postpartum days, parity, dietary indices (macronutritional, phytochemical and energetic), and insomnia, with a Fisher’s post hoc contrast. C1: *n* = 34; C2: *n* = 24; C3: *n* = 17. *d* = Cohen’s d for effect size; * *p* < 0.05.

Figure 5 shows the results for the clusters > 20 mg/d on RAVLT, SCWT, and VFT. In this sense, women from C1 scored lower on forgetting speed and retention when compared with C2, but higher on attentional interference (SCWT). In addition, C1 showed higher means of proactive and retroactive interference than C3. Women from C1 produced significantly fewer words on the VFT letter F than the other ones. The effect sizes were moderate. Regarding adjustment variables, educational level was associated with all of the cognitive measures (*p*-values < 0.05). In addition, parity (primiparous > multiparous) and EDI (β = 0.04) were related, respectively, to Stroop interference and retroactive interference (*p* = 0.008 for both), whilst the number of trials to complete the first category was associated with MDI (β = −1.45, *p* = 0.0059) and EDI (β = 0.84, *p* = 0.0331).

**Figure 6:**
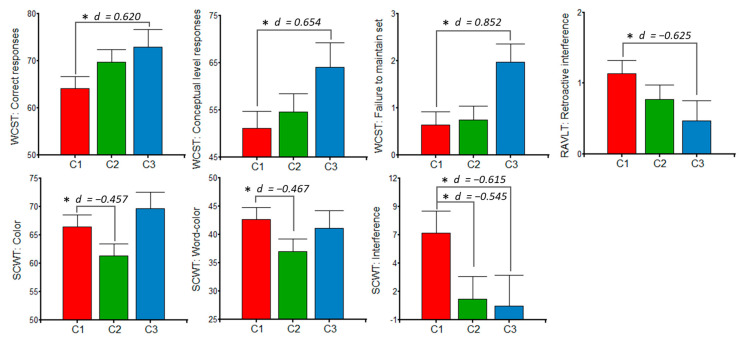
Postpartum women’s performance in the Wisconsin Card Sorting Test (WCST), Rey Auditory Verbal Learning Test (RAVLT), and Stroop Color and Word Test (SCWT) according to polyphenolic intake clusters (5 to 20 mg/d). ANCOVA models were adjusted by educational level, postpartum days, parity, dietary indices (macronutritional, phytochemical, and energetic), and insomnia, with a Fisher’s post hoc contrast. C1: *n* = 33; C2: *n* = 29; C3: *n* = 13. *d* = Cohen’s d for effect size; * *p* < 0.05.

There were also significant differences among the clusters for the polyphenols consumed between 5 to 20 mg/d (Figure 6). In this regard, C1 had lower scores in WCST than C3: correct responses; conceptual level responses; and failure to maintain set. Furthermore, C1 showed higher retroactive interference (RAVLT) and attentional interference (SCWT) than C3. Cluster 1 also differed from C2, which presented lower scores on color and word-color tasks, with a small effect size. Most effect sizes were medium. Concerning adjustment variables, educational level was associated with all of the cognitive measures (*p* < 0.05), parity with Stroop interference (primiparous > multiparous, *p* = 0.007), and EDI with retroactive interference (β = 0.04, *p* = 0.007).

### 3.4. Cognitive Profiles of Postpartum Women

**Figure 7:**
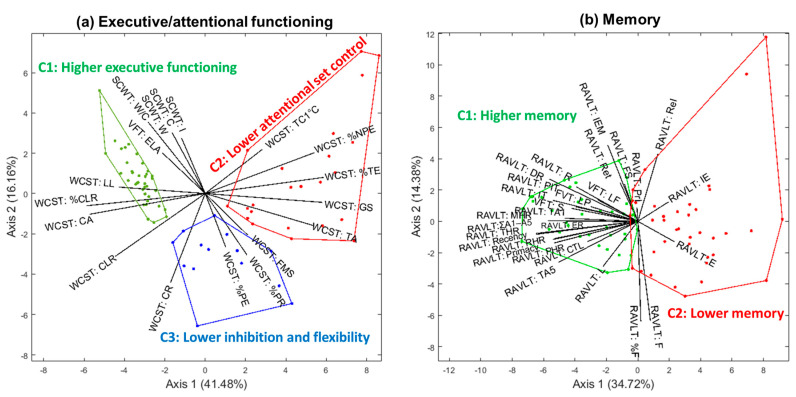
HJ-Biplot representation for clusters in the main plane (axis 1–2) for the executive/attentional functioning (**a**) and memory (**b**) in Argentinian postpartum women. SCWT = Stroop color and word test; I = Interference; C = Color; W = Word; W/C = Word-Color; WCST = Wisconsin card sorting test; TC1 °C = Trials to complete first category; %NPE = Percentage of non-perseverative errors; %TE = Percentage of total errors; GS = Global score; TA = Trials administered; FMS = Failure to maintain set; %PR = Percentage of perseverative responses; %PE = Percentage of perseverative errors; CR = Correct responses; CLR = Conceptual level responses; CA = Categories achieved; %CLR = Percentage of conceptual level responses; LL = Learning to learn; VFT:ELA = Verbal fluency task, excluded letter A; RHR = recency hit rate; MHR = middle hit rate; TA1 = Trial A1; IT = interference trial (B); PIT = post-interference trial (A6); PHR = primacy hit rate; R = recognition; DR = delayed recall; MEI = memory efficiency index; F = forgetting; %F = percentage of forgetting; L = Learning; CTL = Corrected total learning; ΣA1–5 = sum trials A1 to A5; IR, immediate recall; VF-P, verbal fluency letter P; VF-F, verbal fluency letter F; VF-Animals, verbal fluency Animals; PrI = proactive interference; ReI = retroactive interference; TA5 = trial A5; E = evocation; Ret = retention; IE = Intrusion errors; ER = errors of repetitions; FS = forgetting speed; VFT = verbal fluency task; LF = letter F; LP = letter P; S = semantic.

First, the measures of executive/attentional functioning were analyzed together, using the HJ-Biplot technique (Figure 7a). By retaining two axes, the explained variance was 41.48% for the first axis and 16.16% for the second one, which indicated that this model explained over 50% of the data variability. Representation contributions and qualities indicated that the best subspace to interpret results was the plane 1–2, with all of the elements showing good representation (QLR > 200) (Supplementary Materials: Table S3). Three clusters were identified with good quality:

- Cluster 1 or “higher executive/attentional performance”: This conglomerate represented 49.33% (*n* = 37) of the sample and was comprised of women with better scores on positive measures (e.g., conceptual level responses, learning to learn, and categories achieved). This conglomerate had a good quality of representation at the 1–2 plane (QLR = 99%);
- Cluster 2 or “lower attentional set control”: This conglomerate (32.00% of the sample, *n* = 24) was comprised of women with low scoring in the SCWT and with high scores in the negative WCST indices (e.g., global score and trials to complete the first category), and had an adequate quality of representation (QLR = 98%);
- Cluster 3 or “lower inhibition and flexibility”: Fourteen women (18.67%) comprised this cluster, and presented lower scores of inhibitory control and behavioral flexibility in the WCST (i.e., higher perseverative responses, perseverative errors, and failures to maintain set). Its representation quality was good with a QLR of 87%.

Figure 7b shows the HJ-Biplot representation of memory performance. The variance explained by the first axis was 34.72%, and 14.38% by the second one. In addition, contributions and qualities of all of the RAVLT and FVT scores confirmed that this model should be interpreted at the 1–2 plane (Supplementary Materials: Table S4). Two clusters were identified: Cluster 1 or “higher memory”, and Cluster 2 or “lower memory”, which contained 54.67% (*n* = 41) and 45.33% (*n* = 34) of the women, respectively. Both clusters showed a good quality of representation in the plane 1–2 (QLR of 99% for both).

### 3.5. Human Milk Lipids: Effects of Dietary Polyphenols and Cognitive Status

Finally, the ANCOVA models for the human milk lipids according to the polyphenol intake clusters are displayed in Table 5. Levene’s test (*p* > 0.05) indicated that the variances between the groups were statistically equal for all of the chemical variables. Regarding polyphenols between 5 and 20 mg/d, C3 had a significantly higher concentration of triacylglycerols than C1 (30.52 (2.79) versus 23.76 (1.87) g/L, *p* < 0.05, R^2^ = 0.17). In addition, C2 showed higher levels of cholesterol (113.71 (11.46) versus 80.04 (10.85) mg/L, *p* < 0.05, R^2^ = 0.17). These results had medium size effects (Cohen’s d > 0.50). No other significant differences were found. Moreover, no other statistically significant associations were found between polyphenol intake and human milk lipids or adjustment variables.

On the other hand, outcomes revealed that women with higher executive/attentional functioning (Cluster 1) had higher milk concentration of triacylglycerols (28.53 (1.71) g/L) than those with lower inhibition and behavioral flexibility (21.90 (8.84) g/L) and lower attentional set control (22.94 (1.98) g/L) (*p* < 0.05, R^2^ = 0.20). In addition, Cluster 1 showed a lower level of oxidized triacylglycerols (189.17 (103.55) OD/g) when compared with Cluster 3 (640.97 (172.12) OD/g) (*p* < 0.05, R^2^ = 0.22). In respect of women with better memory functioning, they showed more triacylglycerols (27.69 (1.60) g/L vs 22.83 (1.77) g/L, *p* < 0.05, R^2^ = 0.17) and lower oxidized triacylglycerols (194.52 (27.69) OD/g vs. 281.28 (30.85) OD/g, *p* < 0.05, R^2^ = 0.15). As can be seen in Table 6, the effects were medium in size in the executive-attentional functioning model (Cohen’s d > 0.50) and small in the memory model (Cohen’s d > 0.20). Concerning the variables that were included for the adjustment of ANCOVA models, insomnia was negatively related to milk cholesterol content in the executive/attentional functioning model (β = −3.13, *p* = 0.04, R^2^ = 0.22) and the memory model (β = −2.70, *p* = 0.07, R^2^ = 0.17). No other statistically significant associations were found between executive and attentional functioning and human milk lipids or adjustment variables.

Additionally, the analyses of human milk lipids according to extraction time, gestational age at delivery, breastfeeding frequency, and dietary macronutrients are displayed in Table S5 (Supplementary Materials). No associations were found in these ANCOVA models.

## 4. Discussion

Results showed marked interindividual variability in the daily intake of polyphenols, which were associated with better executive/attentional functioning and memory performance in postpartum women. In addition, these dietary compounds significantly correlated with the lipid profile of breast milk, with higher triacylglycerols and cholesterol concentrations. Furthermore, women with better cognitive status showed a better quantity and quality of these nutrients. To the best of our knowledge, this is the first study that integrates the effect of maternal diet and cognition on human milk composition. Taken together, these results support the stated hypothesis.

In this work, we used valid instruments to measure executive/attentional functioning and memory, to include their scores in statistical analyses (Henríquez et al. 2022). Most of them showed a normal distribution, confirming their adequacy for multivariate analyses (Aminu and Shariff 2014).

The nutritional assessment reported that 36 polyphenols presented a mean intake above 5 mg/d. Hydroxycinnamic acids represented the most ingested family, in concordance with previous research about the South American diet. This diet is composed of foods and beverages rich in hydroxycinnamic acids, lignans, and some flavonoids (Carnauba et al. 2021; Rossi et al. 2018).

Nutrition regulates physiological pathways via synergistic nutrient effects (Margină et al. 2020). Polyphenols with cognitive effects were hesperetin, lariciresinol, caffeic acid, and ferulic acid (for those consumed above 20 mg/d) and gallated flavonoids (for those consumed between 5 and 20 mg/d). Women who consumed these compounds showed higher scores in executive function, attention, memory, and language. These results are supported by studies about the cognitive benefits of polyphenols in other populations. In this sense, middle-aged women’s verbal memory is enhanced by the intake of lignans (Greendale et al. 2012), which showed brain bioaccumulation and prefrontal improvement (Kreijkamp-Kaspers et al. 2007; Wang et al. 2018).

On the other hand, flavonoids are neuroprotective by increasing blood flow and strengthening brain pathways related to executive functioning, learning, and memory (Bakoyiannis et al. 2019; Williamson et al. 2018; Kennedy et al. 2017). In the current study, hesperetin, a flavanone, belonged to the cluster associated with better cognitive performance. It was shown that hesperetin, naringenin, and their metabolites, are lipophilic flavonoids that cross hematic barriers (Bakoyiannis et al. 2019). In addition, hesperetin reduces lipid peroxidation (Moghaddam and Zare 2018). In addition to this antioxidant effect, it exerts positive effects on synaptic plasticity in cortical and hippocampal neurons (Vauzour et al. 2007). Only very few clinical trials have established the cognitive effects of hesperetin, which enhances cerebral blood flow and cognition (Salehi et al. 2021).

Only a small amount of information encoded in the nervous system is consolidated and stored by the long-term memory, whereas the rest is forgotten through passive and active mechanisms (Davis and Zhong 2017). In this work, women with low intake of polyphenols presented more interference than women with a higher intake. Memory interference occurs when additional information competes before, after, or during the coding of target information (Crawford et al. 2020). Lariciresinol, hesperetin, and caffeic acid were inversely related to proactive and retroactive interference (moderate effect sizes), with the latter being also associated with gallated flavonoids. Proactive interference occurs when previous knowledge hinders the ability to acquire new information, while retroactive interference refers to the effect of new learning on previously established knowledge, impairing memory consolidation (Crawford et al. 2020). Consequently, retroactive interference is more frequent and harmful (Edwards 2010). There is little evidence about the dietary polyphenolic effect on interference phenomena. Some data show that the intake polyphenolic sources improve memory consolidation by decreasing interference (Bell and Williams 2019) and diet-induced neuroplasticity in postpartum women (Xavier et al. 2021). Other studies found non-significant results (Lamport et al. 2016a; Lamport et al. 2016b) or contradictory ones (Whyte and Williams 2015). Nevertheless, polyphenols attenuate interference and promote cognition and attention (Crawford et al. 2020), which were also improved by these compounds in the present study.

Certain hydroxycinnamic acids formed the cluster with the greatest cognitive outcome, together with hesperetin (flavanone) and lariciresinol (lignan). However, the highly consumed chlorogenic acids, other hydroxycinnamic compounds, were less involved. In terms of cerebral function, there is little evidence about their effects on humans (Coman and Vodnar 2020), with contradictory data (Cropley et al. 2012; Camfield et al. 2013). The cognitive effects of chlorogenic acids imply mood improvement and acetylcholinesterase downregulation (Bouayed et al. 2007; Kwon et al. 2010). This evidence could partially explain the weak outcomes in postpartum women with a higher intake of chlorogenic acids. Conversely, the ferulic and caffeic acids exert well-established biological effects in the brain, supporting the results of the current work. These pro-cognitive effects depend on their higher intestinal absorption, redox-regulating activity, lipoxygenase inhibition, and glial/neuronal protection (Coman and Vodnar 2020; Singh et al. 2021; Habtemariam 2017).

Catechins were related to better cognition. They are classified into gallated and non-gallated catechins, whether they have gallic moieties or not. The gallated ones achieve more bioavailability (Chu and Pang 2018), with epigallocatechin gallate being anxiolytic, anti-amyloidogenic, and a promoter of memory, social cognition, and attention (Wightman et al. 2012). These benefits depend on their effects on brain tissue, such as antioxidant activity, improvement of parietal-frontal connectivity, neuroplasticity, and neuroreceptor modulation (Mancini et al. 2017).

Multiple effects of polyphenols on human milk and child health were described (Ríos et al. 2021). We discovered that these dietary compounds (polyphenols consumed at 5 to 20 mg/d) were positively associated with milk triacylglycerols and cholesterol, with low polyphenol intake being related to milk values below expected averages (Koletzko 2016). Polyphenols modulate lipid metabolism and adipokine secretion (Boccellino and D’Angelo 2020; Carpene et al. 2015). Experimental maternal models found metabolic and genetic effects. In this sense, polyphenols trigger breast tissue-specific gene upregulation, fatty acid bioavailability, lipogenesis, and adiponectin pathway (Caimari et al. 2017). Nonetheless, these mechanisms remain unknown in women, and in human milk.

Triacylglycerols are the main energetic component in human milk (Demmelmair and Koletzko 2018), which is produced by the mammary gland from circulating fatty acids and de novo synthesis to form fat globules (Yang et al. 2018). Numerous factors modify these processes (Yao et al. 2020), such as maternal genetics, environment, and diet (Yang et al. 2018). Adipokines and PPAR regulation determinate lipid homeostasis and inflammatory response to maintain human milk quality (Yang et al. 2018). Moreover, polyphenols target these pathways (Farràs et al. 2013). Thus, the higher lipid level found in women who consumed flavonoids may respond to these complex mechanisms (Caimari et al. 2017; Rabadan-Chávez et al. 2016; McManaman 2014).

The pharmacological potential of polyphenols, such as procyanidin, is applied against dyslipidemia (Rufino et al. 2021; Downing et al. 2017). Feng et al. (2019) also found that epigallocatechin gallate, kaempferol, and quercetin are bioactive on cholesterol metabolism by multiple gene regulation. Moreover, ellagic acid induces lipid metabolism (Kubota et al. 2019), with vegetable-based foods triggering lipid synthesis in breast tissue in an animal model (Palin et al. 2014). Lignans (lariciresinol, pinoresinol) and phenolic acids also contribute (Herchi et al. 2014; Zhong et al. 2021). Quercetin induces sensitivity to prolactin and the expression of stearoyl-CoA desaturase and fatty acid synthase in the murine mammary gland (Lin et al. 2018).

There is growing interest in the effect of psychological factors on breastfeeding (Fallon et al. 2016). However, few studies evaluated the impact of mental health on milk composition (Ziomkiewicz et al. 2021). Our study established that women with better executive/attentional functioning and memory showed a better lipid profile, characterized by increased triacylglycerols with lower oxidation. Ziomkiewicz et al. (2021) found that maternal response to stress is associated with neuroendocrine alterations and breast milk changes. Moreover, chronic stress leads to lipid oxidation (Troubat et al. 2009), with breastfeeding and cortisol levels responding to relaxation interventions (Mohd Shukri et al. 2019).

An inverse association existed between milk cholesterol concentration and insomnia severity. Given that dietary intake and fat storage were considered in multivariate analyses (confounding control), endogenous mechanisms are suggested. Lipids reach human milk from plasma and glandular biosynthesis by insomnia-sensitive mechanisms (Ontsouka and Albrecht 2014; Mohammad and Haymond 2013; Andreas et al. 2015; Fu et al. 2015; Casey et al. 2014). Although cholesterol was within a normal range, our results proposed that severe insomnia could compromise milk composition.

A limitation of this work is that specific mood indicators, which may modify milk composition, were not included (Kawano and Emori 2015; Stuebe et al. 2012). Nevertheless, clinical aspects of mood disorders, such as insomnia, were considered. Another possible limitation can be the sample size and the cross-sectional design. Although potential confounders were adjusted with adequate statistical power, longitudinal studies should replicate this research in larger samples. Although the timing of milk extraction was not significantly associated, milk chronobiology is of interest. Neuropsychological assessment between 8 a.m. and 6 p.m. may cause time-of-day bias (Venkat et al. 2020). Thus, future studies should control this factor. Some of the results have to be interpreted with caution. Future in-depth research should consider collecting milk samples at different time points (i.e., colostrum, transitional milk, and mature milk) with standardized sample collection (e.g., foremilk, hindmilk, complete breast expression, expression time). Specific analyses for human milk lipidomic are also encouraged (e.g., gas chromatography, mass spectrometry, NMR spectroscopy) (George et al. 2018).

Finally, our results reinforce the current perspective to approach puerperal health. Women’s mental health determines self-efficacy, and impacts breastfeeding behaviors and the composition of human milk (Fallon et al. 2016). Nutrient transference to this fluid is essential for the healthy growth and development of children. A complex interaction of psycho-,neuro-,immuno-endocrine responses regulates the maternal brain and mammary gland (Jonas and Woodside 2016).

## 5. Conclusions

In this study, polyphenolic intake during puerperium promoted two relevant aspects of the health of lactating women from Córdoba, Argentina. The first one was that the higher executive/attentional functioning and memory were related to the intake of different families of dietary polyphenols. The second one was a positive association with the milk content of triacylglycerols and cholesterol, crucial lipids for healthy infant growth and development. These findings suggest that human milk lipids can be modulated by certain dietary polyphenols, whose intake of 5–20 mg/d appears to be more suitable for future nutritional interventions. Results, therefore, supported a better cognitive status and lipid profile, which were related. Women with better cognition showed higher triacylglycerols concentration in milk with less peroxidation. In conclusion, maternal polyphenolic intake may improve executive/attentional functioning, memory, and milk lipid profile. Future studies with larger samples, repeated measures, and different methods are needed to validate these findings. The current work constitutes an innovative approach by integrating the assessment of biological, nutritional, and psychological issues of women’s health during this vital stage, and may contribute to understanding the complex nature of human milk.

## Figures and Tables

**Table 1:** Sociodemographic and reproductive characteristics of postpartum women (*n* = 75).

Variable	Mean	SD	%	*N*
Age (years)	29.92	6.13		
Marital status				
In a couple			93.33	70
Single			6.66	5
Educational level				
<12 years of formal instruction			25.33	19
≥12 years of formal instruction			75.66	56
Employment				
Formally employed			45.33	34
Informally employed/unemployed			54.66	41
Postpartum duration (days)	95.04	52.46		
Gestational age at delivery	38.37	2.05		
<37 weeks (preterm)			14.66	11
≥37 weeks (term)			85.33	64
Mode of delivery				
Cesarean section			54.66	41
Vaginal			45.33	34
Number of births				
Primiparous			40	30
Multiparous			60	45
Practice of exclusive breastfeeding				
No			46.66	35
Yes			53.33	40
Breastfeeding frequency (hours)	3.01	2.72		
Time of sampling				
08.00 to 12.00			62.66	47
12.00 to 16.00			18.66	14
16.00 to 20.00			18.66	14
BMI	24.79	5.62		
BFP	28.88	7		
Physical activity (MET)	384.96	686.33		
Macro-nutritional Dietary Index	25.25	6.85		
Phytochemical Dietary Index	21.89	7.18		
Energetic Dietary Index	22.81	7.71		
Fat Quality Index	27.94	23.84		
Protein to Carbohydrate Ratio	0.34	0.08		

Note. SD = standard deviation; BMI = body mass index; BFP = body fat percentage; MET = metabolic equivalents of task.

**Table 2:** Postpartum women’s performance in the Wisconsin Card Sorting Test (*n* = 75).

WCST Score	M	SD	Mdn	25P–75P	S	K
Trials administered	100.67	25.35	93.00	76.00–128.00	0.03	−1.83
Correct responses	67.36	12.28	66.00	62.00–75.00	−0.28	1.10
Total errors	33.30	26.68	20.00	11.00–55.00	0.76	−0.77
% Total errors	29.13	18.44	20.40	14.67–42.97	0.87	−0.45
Perseverative responses	12.64	9.59	9.00	6.00–16.00	1.30	0.67
% Perseverative responses	11.62	6.88	9.33	7.14–14.13	1.09	0.63
Perseverative errors	9.79	7.43	7.00	5.00–12.00	1.33	0.77
% Perseverative errors	11.06	12.59	7.62	5.71–11.69	4.50	21.78
Non-perseverative errors	23.32	23.61	11.00	6.00–37.00	1.32	0.91
% Non-perseverative errors	20.15	18.66	11.68	7.59–28.13	1.80	3.32
Conceptual level responses	55.89	17.68	60.00	54.00–65.00	−1.34	1.65
% Conceptual level responses	61.12	25.80	73.74	42.19–82.28	−0.77	−0.65
Categories achieved	4.49	2.06	6.00	3.00–6.00	−0.95	−0.73
Global score	55.74	43.62	33.00	16.00–98.00	0.37	−1.57
Trials to complete first category	19.13	24.53	11.00	11.00–13.00	3.56	11.35
Failure to maintain set	0.70	1.18	0.00	0.00–1.00	1.91	2.92
Learning to learn	−1.84	4.13	−1.26	−2.86–0.33	−1.50	2.59

Note. M = mean; SD = standard deviation; Mdn = median; 25P = 25th percentile; 75P = 75th percentile; S = Skewness; K = Kurtosis.

**Table 3:** Postpartum women’s performance in the Rey Auditory Verbal Learning Test (*n* = 75).

RAVLT Score	M	SD	Mdn	25P–75P	S	K
Trial A1	5.68	2.02	5.00	4.00–7.00	0.52	0.26
Trial A2	8.03	2.74	8.00	6.00–9.00	0.30	−0.41
Trial A3	9.73	3.09	10.00	8.00–12.00	−0.54	−0.43
Trial A4	10.82	2.72	11.00	9.00–13.00	−1.07	1.15
Trial A5	11.48	2.62	12.00	11.00–13.00	−1.51	3.97
Σ A1–A5	45.95	11.11	48.00	37.00–53.00	−0.17	−0.50
Interference (trial B)	5.39	2.38	5.00	4.00–6.00	0.91	0.81
Post-interference (trial A6)	10.07	2.61	10.00	8.00–12.00	−0.15	−0.50
Delayed recall (trial A7)	10.19	2.86	10.00	9.00–12.00	−0.25	−0.63
Recognition (trial A8)	12.99	2.21	14.00	11.00–15.00	−1.29	1.11
Errors of repetitions	5.45	3.93	5.00	3.00–7.00	1.18	1.46
Intrusion errors	2.89	4.61	1.00	0.00–4.00	3.95	20.01
Corrected total learning	17.34	7.87	18.00	12.00–24.00	−0.56	−0.53
Learning	5.73	2.37	6.00	4.00–7.00	−1.03	2.57
Forgetting	1.31	2.20	1.00	0.00–3.00	−0.79	2.13
% of forgetting	0.12	0.23	0.10	0.00–0.25	−2.69	14.73
Forgetting speed	1.02	0.19	1.00	0.91–1.11	0.27	0.07
Retention	0.05	1.67	0.00	−1.00–1.00	−0.23	−0.20
Evocation	2.63	2.38	3.00	1.00–4.00	−0.70	2.29
Primacy	0.65	0.16	0.65	0.55–0.80	−0.24	−0.83
Recency	0.68	0.16	0.70	0.60–0.80	−0.51	−0.37
Total hit rate	0.59	0.12	0.61	0.49–0.67	−0.08	−0.59
Primacy hit rate	0.62	0.13	0.63	0.54–0.75	−0.19	−0.88
Middle hit rate	0.54	0.16	0.54	0.44–0.68	−0.16	−0.52
Recency hit rate	0.65	0.15	0.67	0.54–0.75	−0.43	−0.54
Memory Efficiency Index	1.99	0.32	2.04	1.84–2.19	−0.44	1.22
Proactive interference	1.03	0.51	1.00	0.71–1.25	1.73	4.31
Retroactive interference	0.90	0.32	0.89	0.75–1.00	4.20	24.40

Note. M = mean; SD = standard deviation; Mdn = median; 25P = 25th percentile; 75P = 75th percentile; S = Skewness; K = Kurtosis.

**Table 4:** Postpartum women’s performance in the Stroop Color and Word Test, Verbal Fluency Tasks, and self-report complaints (*n* = 75).

Scores	M	SD	Mdn	P25–P75	S	K
Stroop Color and Word Test						
Word	92.51	13.93	96.00	85.00–102.00	−0.84	0.39
Color	67.01	10.94	69.00	59.00–74.00	−0.24	−0.17
Word-color	40.50	8.86	41.50	35.00–47.00	−0.47	0.26
Interference	1.87	7.71	1.54	−2.66–6.48	0.10	0.87
Verbal fluency task						
Phonological Letter P	14.03	4.38	13.00	11.00–17.00	0.48	−0.50
Phonological Letter F	9.73	3.62	9.50	7.00–12.00	0.56	0.41
Semantic Animals	17.55	5.68	17.00	14.00–22.00	−0.18	0.25
Phonological Letter Excluded A	7.69	3.90	8.00	5.00–11.00	0.12	−0.03
Self-report complaints						
Insomnia Severity Index	9.78	4.77	9.00	7.00–12.00	0.44	0.10
Executive Complaints	24.64	8.85	25.00	17.00–31.00	0.01	−0.60
Memory Failures of Everyday	28.76	17.27	25.00	16.00–36.00	1.02	0.69

Note. M = mean; SD = standard deviation; Mdn = median; 25P = 25th percentile; 75P = 75th percentile; S = Skewness; K = Kurtosis.

**Table 5:** Human milk lipids’ concentration according to polyphenolic clusters in postpartum women from Córdoba, Argentina (*n* = 75).

Compound	Polyphenols from 5 to 20 mg/d				Polyphenols > 20 mg/d			
	C1 (*n* = 33)	C2 (*n* = 29)	C3 (*n* = 13)	*d*	C1 (*n* = 34)	C2 (*n* = 24)	C3 (*n* = 17)	*d*
Triacylglycerols (g/L)	23.76 (1.87)	25.10 (1.99)	30.52 (2.79) *	0.654	25.06 (2.03)	26.61 (2.19)	24.81 (2.98)	-
Cholesterol (mg/L)	80.04 (10.85)	113.71 (11.46) *	97.73 (16.10)	0.552	85.94 (11.51)	92.02 (16.91)	112.63 (12.38)	-
Oxidized triacylglycerols (OD/g)	335.96 (116.59)	237.66 (123.71)	248.77 (173.78)	-	275.09 (122.65)	241.49 (132.17)	349.39 (180.12)	-
Polar oxysterols (OD/mg)	92.90 (10.77)	80.39 (11.37)	84.81 (15.97)	-	91.79 (11.28)	78.72 (12.13)	88.18 (16.57)	-
Non-polar oxysterols (OD/mg)	81.31 (8.50)	59.02 (8.97)	61.65 (12.61)	-	71.11 (9.11)	62.43 (9.80)	74.85 (13.38)	-

Note. Data expressed as mean (standard error). ANCOVA models were adjusted by breastfeeding, parity, postpartum days, body fat percentage, and dietary indices (macronutritional, phytochemical and energetic), with post hoc comparisons (*d* = Cohen’s d for size effect; * *p* < 0.05). Thresholds of the effect size: small (0.20), medium (0.50), large (0.80), very large (1.30).

**Table 6:** Human milk lipids concentration according to executive/attentional functioning and memory of postpartum women from Córdoba, Argentina (*n* = 75).

Compound	Executive/Attentional Functioning				Memory		
	>EF (*n* = 37)	<IF (*n* = 14)	<AS (*n* = 24)	*d*	>M (*n* = 41)	<M (*n* = 34)	*d*
Triacylglycerols (g/L)	28.53 (1.71)	21.90 (2.84) *	22.94 (1.98) *	−0.647 −0.561	27.69 (1.60)	22.83 (1.77) *	−0.479
Cholesterol (mg/L)	90.97 (10.26)	112.34 (17.88)	96.84 (11.88)	-	94.35 (9.53)	98.36 (10.70)	-
Oxidized triacylglycerols (OD/g)	189.17 (103.55)	640.97 (172.12) *	241.97 (120.01)	0.727	194.52 (27.69)	281.28 (30.85) *	0.493
Polar oxysterols (OD/mg)	95.34 (9.77)	65.13 (17.03)	84.06 (11.32)	-	92.89 (9.10)	78.89 (10.22)	-
Non-polar oxysterols (OD/mg)	71.32 (7.97)	55.70 (13.90)	72.32 (9.24)	-	64.96 (7.37)	74.48 (8.28)	-

Note. Data expressed as mean (standard error). ANCOVA models were adjusted by breastfeeding, parity, postpartum days, body fat percentage, dietary indices (macronutritional, phytochemical, and energetic), and insomnia severity index, with post hoc comparisons (*d* = Cohen’s d for size effect; * *p* < 0.05). Thresholds of the effect size: small (0.20), medium (0.50), large (0.80), very large (1.30). >EF = cluster with higher executive functioning; <IF = cluster with lower inhibitory control and behavioral flexibility; <AS = cluster with lower attentional set control; M = memory.

## Data Availability

The data presented in this study are available on request from the corresponding author. The data are not publicly available due to ethical restrictions.

## Supplementary Materials

The following supporting information can be downloaded at: https://www.mdpi.com/article/10.3390/jintelligence10020033/s1, Table S1: Contributions and representation qualities for each polyphenol consumed above 20 mg/d on the first two axes; Table S2: Contributions and representation qualities for each polyphenol consumed from 5 to 20 mg/d on the first two axes; Table S3: Contributions and representation qualities for each executive/attentional score on the first two axes; Table S4: Contributions and representation qualities for each memory score on the first two axes; Table S5: Human milk lipids according to extraction time, gestational age at delivery, breastfeeding frequency, and dietary macronutrients.
