# Supplementary material for: Dietary Intake of Polyphenols Enhances Executive/Attentional Functioning and Memory with an Improvement of the Milk Lipid Profile of Postpartum Women from Argentina
Source: J Intell. 2022 May 31;10(2):33. doi: 10.3390/jintelligence10020033 (PMC9224741; doi:10.3390/jintelligence10020033)
Supplement: Supplementary file 1 [file jintelligence-10-00033-s001.zip › jintelligence-1665521-supplementary.pdf]

Table S1. Contributions and representation qualities for each polyphenol consumed above 20 mg/d on the first two axes.

	Contribution		QRL	
**Polyphenol (mg/d)**	**Axis 1**	**Axis 2**	**Axis 1**	**Axis 2**
Hesperetin	7	235	7	242
Quercetin 3–O–rutinoside	945	35	945	980
1–Caffeoylquinic acid	931	60	931	991
1,3–Dicaffeoylquinic acid	931	60	931	991
1,4–Dicaffeoylquinic acid	931	60	931	991
3,4–Dicaffeoylquinic acid	968	28	968	996
3–Caffeoylquinic acid	803	112	803	915
4,5–Dicaffeoylquinic acid	931	60	931	991
4–Caffeoylquinic acid	748	133	748	881
5–Caffeoylquinic acid	849	98	849	947
Caffeic acid	26	703	26	729
Ferulic acid	20	224	20	244
Lariciresinol	54	357	54	411

Note. The axes establish the HJ-Biplot reference system and represent latent factorial variables obtained from linear combinations of the initially observed variables. QLR = Quality of representation.

Table S2. Contributions and representation qualities for each polyphenol consumed from 5 to 20 mg/d on the first two axes.

	Contribution		QLR	
**Polyphenol (mg/d)**	**Axis 1**	**Axis 2**	**Axis 1**	**Axis 2**
Malvidin 3-O-glucoside	275	116	275	391
(−)–Epicatechin	621	15	621	636
(−)– Epigallocatechin	691	146	691	837
(−)– Epigallocatechin 3–O–gallate	687	147	687	834
(−)–Epicatechin 3–O–gallate	731	126	731	857
(+)–Catechin	655	67	655	722
(+)–Gallocatechin	689	146	689	835
Procyanidin dimer B2	321	54	321	375
Eriodyctiol	75	2	75	77
Naringenin	0	80	0	80
Quercetin	632	68	632	700
5–O–Galloylquinic acid	687	148	687	835
Ellagic acid	90	57	90	147
Syringic acid	63	77	63	140
Disuccinoylquinic acid	10	484	10	494
3–Feruloylquinic acid	0	656	0	656
4–Feruloylquinic acid	33	743	33	776
5–Feruloylquinic acid	38	734	38	772
Caffeoyl–glucose	10	484	10	494
o–Coumaric acid	69	122	69	191
p–Coumaric acid	145	281	145	426
Trans–ferulic acid	13	2	13	15
Pinoresinol	79	175	79	254

Note. The axes establish the HJ-Biplot reference system and represent latent factorial variables obtained from linear combinations of the initially observed variables. QLR = Quality of representation.

Table S3. Contributions and representation qualities for each executive/attentional score on the first two axes.

	**Contribution**		**QLR**	
**Scores**	**Axis 1**	**Axis 2**	**Axis 1**	**Axis 2**
WCST: Trials administered	783	69	783	852
WCST: Correct responses	81	536	81	617
WCST: % Total errors	953	18	953	971
WCST: % Perseverative responses	118	257	118	375
WCST: % Perseverative errors	28	194	28	222
WCST: % Non-perseverative errors	804	113	804	917
WCST: Conceptual level responses	548	269	548	817
WCST: % Conceptual level responses	952	10	952	962
WCST: Categories achieved	900	29	900	929
WCST: Global score	927	5	927	932
WCST: Trials to complete first category	220	132	220	352
WCST: Failure to maintain set	122	117	122	239
WCST: Learning to learn	505	3	505	508
SCWT: Word	75	254	75	329
SCWT: Color	68	233	68	301
SCWT: Word-color	220	347	220	567
SCWT: Interference	38	208	38	246
FVT: Phonological, Letter Excluded A	124	113	124	237

Note. The axes establish the HJ-Biplot reference system and represent latent factorial variables obtained from linear combinations of the initially observed variables. QLR = Quality of representation; WCST = Wisconsin Card Sorting Test; SCWT = Stroop Color Word Test; FVT = Verbal Fluency Task.

Table S4. Contributions and representation qualities for each memory score on the first two axes.

	Contribution		QLR	
**Score**	**Axis 1**	**Axis 2**	**Axis 1**	**Axis 2**
RAVLT: Trial A1	480	1	480	481
RAVLT: Trial A5	565	152	565	717
RAVLT: Σ A1–A5	932	13	932	945
RAVLT: Interference (trial B)	398	15	398	413
RAVLT: Post-interference (trial A6)	647	40	647	687
RAVLT: Delayed recall (trial A7)	703	161	703	864
RAVLT: Recognition (trial A8)	337	107	337	444
RAVLT: Errors of repetitions	58	0	58	58
RAVLT: Intrusion errors	84	34	84	118
RAVLT: Corrected total learning	109	185	109	294
RAVLT: Learning	227	37	227	264
RAVLT: Forgetting	14	885	14	899
RAVLT: % of forgetting	1	903	1	904
RAVLT: Forgetting speed	16	125	16	141
RAVLT: Retention	28	134	28	162
RAVLT: Evocation	121	38	121	159
RAVLT: Primacy	630	42	630	672
RAVLT: Recency	645	30	645	675
RAVLT: Total hit rate	928	15	928	943
RAVLT: Primacy hit rate	633	43	633	676
RAVLT: Middle hit rate	731	0	731	731
RAVLT: Recency hit rate	627	27	627	654
RAVLT: Memory Efficiency Index	82	528	82	610
RAVLT: Proactive interference	0	17	0	17
RAVLT: Retroactive interference	36	411	36	447
VFT: Phonological, Letter P	198	20	198	218
VFT: Phonological, Letter F	145	42	145	187
VFT: Semantic, Animals	347	19	347	366

Note. The axes establish the HJ-Biplot reference system and represent latent factorial variables obtained from linear combinations of the initially observed variables. QLR = Quality of representation; RAVLT = Rey Auditory Verbal Learning Test; VFT = Verbal Fluency Task.

Table S5. Human milk lipids according to extraction time, gestational age at delivery, breastfeeding frequency, and dietary macronutrients.

	**TAG (g/L)**		**Chol (g/L)**		**OTAG (OD/g)**		**PO (OD/mg)**		**NPO (OD/mg)**	
	**F**	***p***	**F**	***p***	**F**	***p***	**F**	***p***	**F**	***p***
Milk extraction time	0.61	0.5470	0.67	0.5168	0.16	0.8519	0.93	0.4023	1.96	0.1508
Gestational age at delivery	1.86	0.1780	0.13	0.7151	0.38	0.5384	0.54	0.4651	0.31	0.5796
Fat quality index	0.16	0.6879	0.19	0.6632	0.07	0.7970	0.73	0.3962	0.01	0.9115
Protein to carbohydrate ratio	0.00	0.9934	3.02	0.0883	3.53	0.0658	0.19	0.6644	2.41	0.1264
Breastfeeding frequency	0.08	0.7831	0.16	0.6945	0.04	0.8458	0.00	0.9782	1.96	0.1678

Note. TAG = triacylglycerols, Chol = cholesterol; OTAG = oxidized triacylglycerols; PO = polar oxysterols; NPO = non-polar oxysterols. F = F-value of ANCOVA.
